# Moderate-coherence sensing with optical cavities: ultra-high accuracy meets ultra-high measurement bandwidth and range

**DOI:** 10.1038/s44172-024-00164-w

**Published:** 2024-01-25

**Authors:** Johannes Dickmann, Liam Shelling Neto, Steffen Sauer, Stefanie Kroker

**Affiliations:** 1 CAVITY technologies UG (haftungsbeschränkt), Wilhelmsgarten 3, 38100 Braunschweig, Germany; 2 Technical University of Braunschweig, Institute for Semiconductor Technology, Hans-Sommer-Str. 66, 38106 Braunschweig, Germany; 3 Laboratory for Emerging Nanometrology (LENA), Langer Kamp 6a/b, 38106 Braunschweig, Germany; 4 Physikalisch-Technische Bundesanstalt, Bundesallee 100, 38116 Braunschweig, Germany

**Keywords:** Aerospace engineering, Optical techniques, Mechanical engineering, Optical sensors

## Abstract

Interferometric sensors, renowned for their exceptional accuracy, leverage the wave properties of coherent electromagnetic radiation. The periodicity of the measurement signal often critically limits the measurement range of sensors utilizing interferometry. Here we introduce a cavity-based interferometry concept that capitalizes on a laser with moderate coherence, thereby combining ultra-high accuracy with ultra-high measurement bandwidth and range. To this end mid-fringe detection is combined with measurements of the interferometric visibility. We present experimental results that demonstrate the effectiveness of our approach exemplarily for length sensing. Notably, our system achieves an accuracy of 1 nm with a measurement range of 120 μm (relative uncertainty of 0.00083 %) and a bandwidth ranging from 0 Hz to 20 kHz. These findings support advancements in high-precision sensing applications that demand simultaneous accuracy, measurement range and bandwidth.

## Abstract (web summary)

Dickmann and colleagues present an interferometric sensing scheme that delivers improved accuracy with wider dynamic range and high speed. They use the multiple modes of a Fabry-Perot laser diode with the weighted power to measure with moderate coherence and thus break up the periodicity of the sensor signal.

## Introduction

In the field of sensing and metrology, achieving a balance between accuracy and measurement range has been a long-standing challenge. Interferometric sensors, which rely on the wave properties of coherent electromagnetic radiation, have been at the forefront of high-precision measurements^1,2^. However, the exceptional accuracy of interferometric sensors often comes at the cost of a limited range due to the inherent periodicity of the interferometer signal. Interferometric gravitational wave detectors have already demonstrated remarkable precision levels better than 10^−21^ (ref. ^3^), while ultrastable lasers exhibit precision on the order of 10^−17^ (refs. ^4,5^). However, the limited range has hindered their practical utility, preventing them from capturing rapid changes or transient events. A combination of high accuracy in large measurement ranges is also critical for various other fields including precision manufacturing^6–8^, biomedical sensing^9–11^, and structural health monitoring^12,13^.

This research paper introduces a concept called moderate-coherence sensing, which addresses the critical limitation of range in interferometric sensors while preserving their ultra-high accuracy capabilities. By capitalizing on the limited coherence of the measurement laser^14^, this approach offers the potential to combine high accuracy with ultra-high measurement range, enabling advancements in high-precision sensing applications. This paper showcases the experimental implementation of moderate-coherence sensing and presents measurement results highlighting its effectiveness. The system developed achieves an impressive accuracy of 1 nanometer while maintaining a range of 120 micrometers. By overcoming the long-standing limitations of interferometric sensors, moderate-coherence sensing holds the promise of transforming industries and advancing scientific endeavors that rely on both ultra-high precision, wide range, and high-speed capabilities.

## Methods

### Theoretical description

The high precision of interferometric sensors is attributed to the short wavelength of the electromagnetic radiation used in the micrometer (μm) range and the wavelength’s high stability. The Michelson interferometer^15^, as a simple example, demonstrates this precision through the appearance of interference fringes. Consequently, with appropriate laser and readout electronics, sub-nanometer accuracy in length measurements can be easily achieved^15^. However, the periodic nature of the interference signal, with a period equal to half the wavelength, limits the achievable measurement range. Active methods, such as using a movable mirror controlled by a controller, have been employed to expand the range^16^. Nonetheless, these approaches introduce complexity and are constrained by the maximum control speed thereby limiting the measurement bandwidth.

**Fig. 1:**
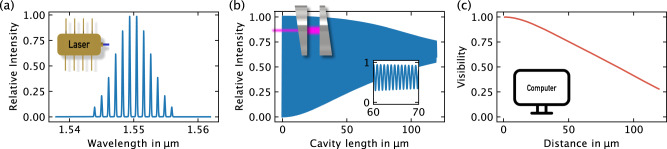
Theoretical description of moderate-coherence sensing. **a** A Fabry-Pérot laser diode generates multiple quasi-monochromatic output wavelengths weighted by the medium gain spectrum. **b** A low-finesse Fabry-Pérot cavity is formed using two wedged silicon wafers for length measurement. **c** The calculated visibility is utilized to disrupt the periodicity of the interference signal.

Our method overcomes the limitations of complex and potentially slow active control while maintaining both high accuracy and a wide range. The key principle lies in leveraging not only the sensitive interference signal of the interferometer but also the limited coherence of the laser employed. Specifically, we utilize a Fabry-Pérot laser diode, which is actively temperature-stabilized to maintain a constant wavelength. Figure 1a illustrates the laser’s emission characteristics, where multiple Fabry-Pérot modes are excited, and their output power is weighted by the Gaussian medium gain spectrum. The width of this spectrum inherently restricts the overall coherence of the laser output.

#### General description

To achieve precise length measurements, we employ a low-finesse Fabry-Pérot cavity comprising two wedged silicon wafers. At the laser wavelength of 1.55 μm, the refractive index of silicon is *n* = 3.4757^17^. Consequently, the intensity reflection of the cavity mirrors is *R* = 30.6 %^18^, corresponding to a Lorentzian finesse of 2.65^19^. To calculate the reflected power of the cavity, we superimpose the individual quasi-coherent emission lines of the laser.

For the calculation, we start by expressing the laser gain:

1 $$G(\lambda )={G}_{0}\exp \left(-\frac{{(\lambda -{\lambda }_{{{{{{{{\rm{center}}}}}}}}})}^{2}}{\Delta {\lambda }_{{{{{{{{\rm{FPL}}}}}}}}}^{2}}\right),$$

where *λ*_center_ = 1.55 μm is the gain center wavelength and Δ*λ*_FPL_ is the gain linewidth. Next, for each emitted mode indexed by *i* with wavelength *λ*_*i*_, we calculate the roundtrip single-pass phase *ϕ*_*i*_ as a function of the cavity length *L*^19^:

2 $${\phi }_{i}(L)=2\pi \frac{L}{{\lambda }_{i}}.$$

Using these parameters, we can determine the power transmitted through the cavity for each mode *i*^19^:

3 $${I}_{i}^{{{{{{{{\rm{trans}}}}}}}}}=\frac{{(1-R)}^{2}}{{(1-R)}^{2}+4R{\sin }^{2}{\phi }_{i}}\times G({\lambda }_{i}).$$

Finally, we obtain the measurand, which is the total power reflected by all modes:

4 $${I}^{{{{{{{{\rm{refl}}}}}}}}}=1-\mathop{\sum }\limits_{i=-\infty }^{\infty }{I}_{i}^{{{{{{{{\rm{trans}}}}}}}}}.$$

The results of the calculation are presented in Fig. 1b, where the classical quasiperiodic interference signal of the cavity is observed. Notably, the periodicity of the signal is disrupted by decreasing visibility. The visibility *V*, quantified by the relative ratio of the maximum ${I}^{\max }$ and minimum ${I}^{\min }$ power values of the fringes, is defined as:

5 $$V(L)=\frac{{I}_{j}^{\max }-{I}_{j}^{\min }}{{I}_{j}^{\max }}$$

for a certain fringe *j*. Figure 1c displays the calculated visibility as a function of the cavity length. A distinctive trend is observed, where an almost linear curve is evident within the range of 10 to 120 μm.

Indeed, the combination of the high-sensitivity interference signal and the utilization of visibility plays a critical role in achieving high accuracy with a wide range of length measurements while achieving measurement precisions down to sub-nanometer levels.

#### Influence of the laser parameters

**Fig. 2:**
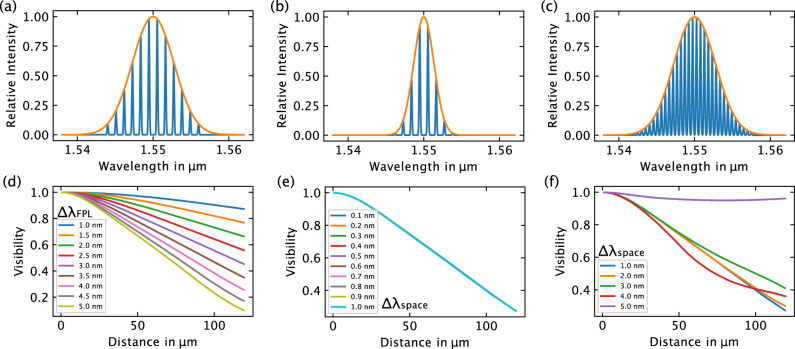
Numerical calculation of expected visibility as a function of critical laser parameters. The width of the Fabry-Pérot laser (FPL) gain, Δ*λ*_FPL_, and the spacing of the laser lines, Δ*λ*_space_. **a** illustrates the laser spectrum with Δ*λ*_FPL_ = 3.9 nm and Δ*λ*_space_ = 1 nm. In **b**, the laser spectrum is depicted for a narrower laser gain (Δ*λ*_FPL_ = 1.9 nm) with Δ*λ*_space_ = 1 nm. **c** demonstrates the laser spectrum for a reduced laser line spacing (Δ*λ*_space_ = 0.5 nm) and Δ*λ*_FPL_ = 3.9 nm. Figure **d** displays the numerically calculated visibility for varying laser gain widths from Δ*λ*_FPL_ = 1 nm to Δ*λ*_FPL_ = 5 nm, maintaining a laser line spacing of Δ*λ*_space_ = 0.5 nm. The observed trend indicates that the decrease in visibility magnifies with widening laser gain. **e** presents the numerically calculated visibility for different laser line spacings (Δ*λ*_space_) ranging from Δ*λ*_space_ = 0.1 nm to 1 nm while keeping Δ*λ*_FPL_ = 3.9 nm constant. No substantial difference is observed. Finally, **f** shows the numerically calculated visibility for diverse laser line spacings between Δ*λ*_space_ = 1 nm and 5 nm at Δ*λ*_FPL_ = 3.9 nm. The nearly linear progression diminishes as Δ*λ*_space_ becomes excessively large.

The expected interferometer signal is calculated based on defined parameters: an average laser wavelength of *λ*_center_ = 1.55 μm and a cavity mirror reflection of *R* = 30.6 %. The width of the laser gain, Δ*λ*_FPL_, and the spacing of individual laser lines, Δ*λ*_space_ = *λ*_*i*+1_ − *λ*_*i*_, are considered as free parameters. A series of numerical simulations explored the dependency of visibility on these parameters. Utilizing equations (1) to (5), the visibility of the interferometer signal was numerically calculated for various combinations of Δ*λ*_FPL_ and Δ*λ*_space_. Figure 2 illustrates these results.

Figure 2a–c visually depicts the influence of these parameters on the laser wavelength spectrum. Figure 2a represents the initial spectrum with Δ*λ*_FPL_ = 3.9 nm and Δ*λ*_space_ = 1 nm. In 2b, the width of the laser gain was reduced to Δ*λ*_FPL_ = 1.9 nm, while the laser line spacing remained constant. Figure 2c showcases the reduction of the laser line spacing to Δ*λ*_space_ = 0.5 nm, while maintaining the original laser gain width at Δ*λ*_FPL_ = 3.9 nm.

Figure 2d demonstrates the dependence of calculated visibility *V* on the width of the laser gain medium, Δ*λ*_FPL_, varying between 1 nm and 5 nm with a fixed laser line spacing of Δ*λ*_space_ = 0.5 nm. The observed trend indicates that greater visibility increases are associated with wider laser gains, attributed to decreasing coherence.

In Fig. 2e, the visibility dependence on laser line spacing, Δ*λ*_space_, is shown for a constant laser gain width of Δ*λ*_FPL_ = 3.9 nm. Varying the laser line spacing between 0.1 nm and 1 nm reveals that, within this range, this parameter has negligible influence on visibility. It is inferred that, with a sufficient number of laser lines in the gain medium, individual lines become indistinguishable. To test this hypothesis, the laser line spacing was increased, resulting in a limited number of lines within the gain width. The results in Fig. 2f highlight a deviation from ideal linear behavior as the laser line spacing approaches the magnitude of the laser medium gain width. This underscores the conclusion that for optimal system functionality, Δ*λ*_space_ ≪ Δ*λ*_FPL_ must be fulfilled.

**Fig. 3:**
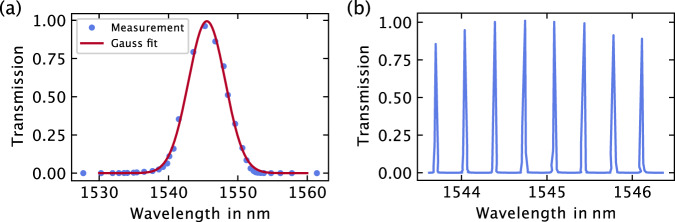
Measurement results of the laser’s transmission spectrum. **a** depicts the gain medium measurement, featuring a well-fitted Gaussian distribution with excellent agreement (*R*^2 ^= 0.985). **b** illustrates a high-resolution wavelength scan of the laser lines, showcasing a spacing of Δ*λ*_space_ = 0.34 nm.

The laser’s emission spectrum was analyzed using an optical spectrum analyzer (OSA), and the results are depicted in Fig. 3. In Fig. 3a, a comprehensive scan across the laser gain medium envelope is presented, with the data fit to equation (1). The derived laser parameters include:

6 $${G}_{0}=1\qquad \,{{\mbox{(normalized measurement)}}}\,,$$

7 $$\Delta {\lambda }_{{{{{{{{\rm{FSR}}}}}}}}}=(3.82\pm 0.08)\,\,{{\mbox{nm}}}\,.$$

Figure 3b illustrates a high-resolution scan encompassing eight laser modes, revealing a mode spacing of:

8 $$\Delta {\lambda }_{{{{{{{{\rm{space}}}}}}}}}=(0.35\pm 0.02)\,\,{{\mbox{nm}}}\,.$$

Notably, this satisfies the crucial condition Δ*λ*_space_ ≪ Δ*λ*_FSR_, rendering the laser highly suitable for the coherence-moderated sensing approach presented in this study.

### Optical sensing scheme

**Fig. 4:**
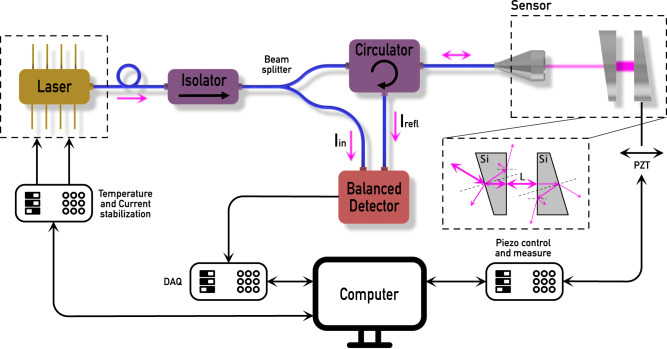
Schematic of the experimental implementation. A temperature and current-stabilized laser passes an isolator and a circulator before coupling to the low-finesse cavity. The cavity length is tuned by a piezoelectric transducer (PZT). The input and reflected laser powers are measured using a data acquisition card (DAQ).

Figure 4 illustrates the experimental implementation of moderate-coherence sensing. This setup comprises several components and instruments to ensure stable and accurate measurements. The system’s core component includes the Fabry-Pérot laser diode (Thorlabs FPL1009S), which is both temperature and current stabilized using the Thorlabs CLD1015 module. The most critical parameter for accuracy is the temperature of the laser diode. According to the manufacturer, the wavelength increases by approx. 0.3 nm per Kelvin. The absolute temperature stabilization is 0.01 K in our setup, which corresponds to a maximum fluctuation of the laser wavelength of 3 pm. This wavelength error leads to a maximum measurement error of 0.23 nm for the full 120 μm measurement bandwidth.

The emitted laser modes are protected from back reflections by passing through a Faraday isolator (Thorlabs IOT-H-1550A) and are then coupled into a single-mode fiber for transmission.

To provide a reference signal, a portion of the light is directed toward a balanced detector (Thorlabs PDB450C) via a fiber beam splitter (Thorlabs TN1550R3A1). The remaining light passes through a circulator (Thorlabs 6015-3-APC) before being coupled out of the fiber using a fiber connector (Thorlabs F280APC-1550).

For length measurement, a low-finesse cavity is constructed using two wedged silicon wafers (Thorlabs WW80530). The first mirror is permanently mounted on the optical table, while the second mirror is positioned on a linear stage (Thorlabs NFL5DP20S/M). The linear stage offers manual movement capability of up to 5 mm and can be piezo-driven with a range of 20 μm. Additionally, the linear stage is equipped with a strain gauge featuring a theoretical resolution of 0.6 nm, allowing for precise cavity length measurement.

However, it should be noted that the travel range of the piezo-driven linear stage is insufficient to fully exploit the 120 μm range of the moderate-coherence sensing technique developed in this study. As a result, additional manual translation was necessary to cover an even wider range. Moreover, as demonstrated later in the paper, the measurement method presented herein exhibits superior accuracy and speed compared to the strain gauge of the linear stage.

The experimental setup described above provides the necessary infrastructure to implement moderate-coherence sensing and carry out the measurements described in the subsequent sections.

## Results

The measurement signal recording was carried out in a semi-automatic manner. Initially, a manual offset of the cavity length ranging from 0 to 100 μm was set. Subsequently, the automated measurement process was initiated. To achieve this, a Python program controlled the piezo stage, enabling the scanning of the entire 0 to 20 μm range in increments of 2 mV (approximately 0.6 nm). After each step, the cavity length was measured using the strain gauge.

Simultaneously, the measurement signal from the moderate-coherence sensing and the optical reference signal were recorded using a measurement card (National Instruments NI USB-6363). This setup facilitated data acquisition at each piezo step, allowing for a comprehensive analysis of the measurement results.

**Fig. 5:**
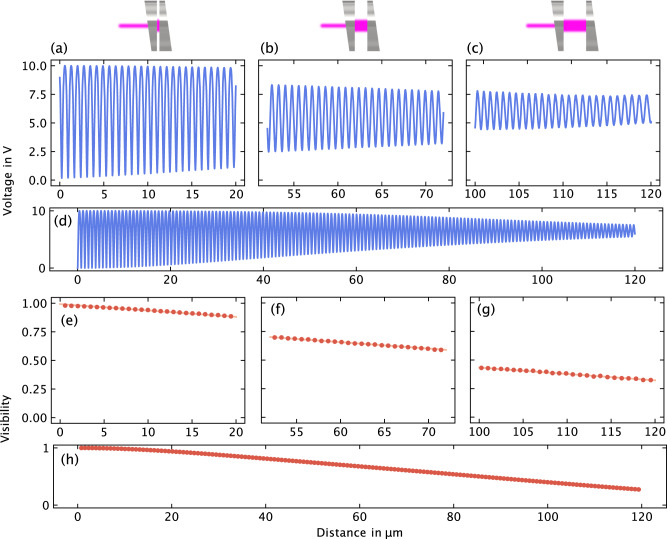
Measurement results of moderate-coherence sensing. **a**–**d** displays the raw signal of the photodetector (voltage) for three distinct sections of the cavity length: 0 to 20 μm, 50 to 70 μm, and 100 to 120 μm as well as for the whole 120 μm range. **e**–**h** presents the calculated visibility derived from the raw data, represented by red dots, along with the theoretical calculation of the visibility shown as a red line. Remarkably, the data exhibit good agreement between the measured and theoretical visibilities, confirming the accuracy and reliability of the moderate-coherence sensing technique.

Figure 5a–d presents the obtained measurement results for a cavity length range spanning from 0 to 120 μm. The plot shows the voltage signal of the photodetector alongside the visibility, calculated from the raw signal using eq. (5), as a function of length (Fig. 5e–h). Additionally, the theoretically calculated visibility (cf. Fig. 1) is illustrated as a solid red line. Notably, the measurement results closely align with the theoretical predictions, with virtually no visually detectable deviations. This excellent agreement demonstrates the dominant and highly measurable nature of the calculated effect in the experiment. Furthermore, it indicates that other factors such as misalignment and finite laser divergence, which were not explicitly considered, can be reasonably neglected in this context. In the following section, we will illustrate how the cavity length can be derived using moderate-coherence sensing in real-time measurements.

### Real-time measurement

In this section, we will explore the practical application of moderate-coherence sensing for real-time length measurements. Once the command for an actual measurement is initiated, the moderate-coherence sensing system rapidly scans the cavity length over half a wavelength, which corresponds to approximately 0.8 μm. This scanning is achieved using a ring piezo integrated into the sensor, modulating the actual cavity length (Model Thorlabs PA44M3KW). The resonant frequency of the piezo used with the load of the silicon mirror (mass 0.9 g) is approx. 150 kHz. Therefore, a scan with the frequency of 20 kHz used here is possible. The maximum displacement of this piezo is 3.9 μm.

**Fig. 6:**
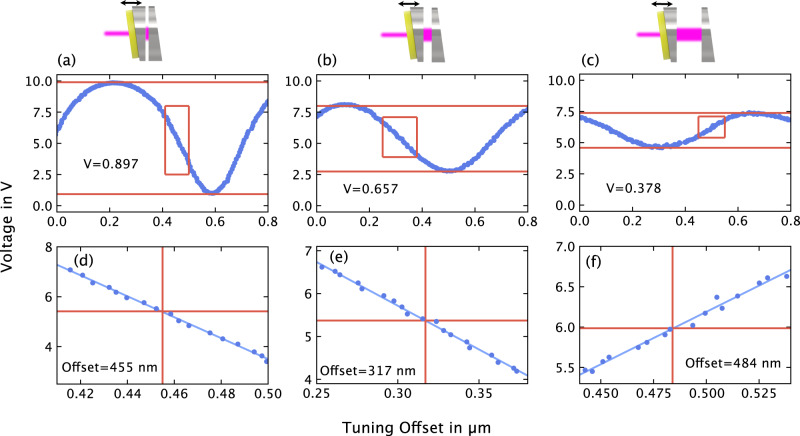
Real-time measurement results. **a**–**c** illustrates the fast piezoscan spanning 0.8 μm and the corresponding calculated visibility (*V*). **d**–**f** displays the filtered data focused on the half fringe (blue dots), the linear regression analysis (blue line), and the calculated relative position of the half fringe (offset).

During the scanning process, at least one fringe maximum and one fringe minimum are captured, as shown in Fig. 6a–c. These data provide the necessary information to calculate the actual cavity length using software algorithms. Firstly, the visibility can be calculated, providing a value for the cavity length with a resolution of 0.3875 μm. Secondly, a section of the recorded data, specifically the mid-fringe, is extracted and filtered (Fig. 6d–f). The mid-fringe is then adjusted using linear regression, enabling precise calculation of its position relative to the original measured cavity length, with an accuracy better than 1 nm.

Since the theoretical position of the mid-fringe is known from the earlier theoretical description (see section “Theoretical description"), the precise cavity length can be calculated accordingly using the following equation:

9 $${L}^{{{{{{{{\rm{meas}}}}}}}}}={L}^{{{{{{{{\rm{vis}}}}}}}}}-\,{{\mbox{Offset}}}\,.$$

**Fig. 7:**
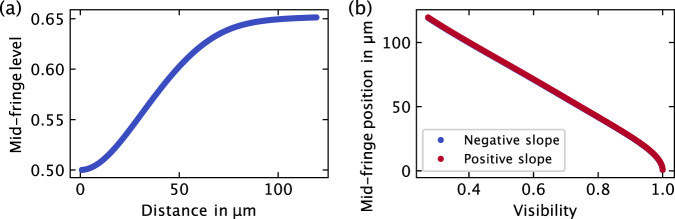
Numerical calculation results depicting the mid-fringe position relative to the signal visibility. In Figure **a**, the relative mid-fringe level is calculated using equations (1)–(8). Figure **b** illustrates the mid-fringe position’s dependency on visibility for both negative and positive mid-fringe increments. The corresponding lookup table is available in the Supplementary Materials.

The numerically calculated mid-fringe position (see Fig. 7a, b) for the measured visibility (see Fig. 6) is denoted as *L*^vis^. This mid-fringe position can be obtained from the lookup table for both negative and positive mid-fringe increments, available in the Supplementary materials (Table 1). The offset is determined through linear regression. For the measurements in Fig. 6, the results are as follows:

1. Visibility *V* = 0.897, negative mid-fringe. Using the lookup table, this corresponds to *L*^vis^ = 26.988 μm. Linear regression yields an offset of 455 nm. Consequently, the measurement, according to equation (9), results in *L*^meas^ = 26.533 μm.
2. Visibility *V* = 0.657, negative mid-fringe →  *L*^vis^ = 62.603 μm, Offset = 317 nm →  *L*^meas^ = 62.286 μm.
3. Visibility *V* = 0.378, positive mid-fringe →  *L*^vis^ = 103.266 μm, Offset = 484 nm →  *L*^meas^ = 102.782 μm.

Following the demonstration of real-time measurement, the accuracy and reproducibility of the moderate-coherence sensing presented here will now be assessed.

### Determination of accuracy and reproducibility

**Fig. 8:**
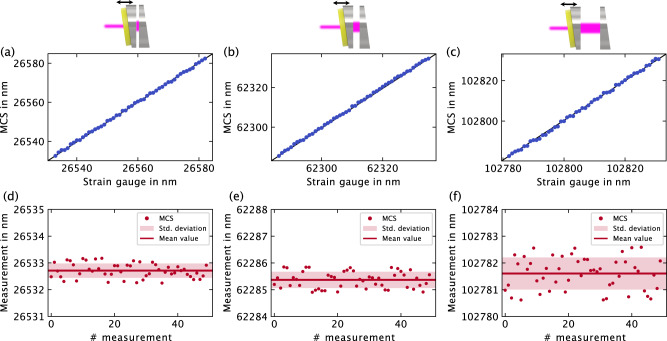
Experimental assessment of accuracy and reproducibility. Figures **a**–**c** depict results from the nanometer-step experiments, plotting moderate-coherence sensing (MCS) measurements against the integrated strain gauge readings of the piezo stage. Figures **d**–**f** represent the repetitive measurements (*n* = 50) of the same cavity length, displaying mean values and standard deviations.

The accuracy and reproducibility of moderate-coherence sensing were assessed for three measurement positions (26.533 μm, 62.286 μm, and 102.782 μm). A nanometer-step experiment was conducted, involving fifty 1 nm steps for each initial position using the piezo stage (Thorlabs NFL5DP20S/M) controlled by the internal strain gauge. Real-time measurements were taken after each step, as described in the preceding section. The results in Fig. 8a–c demonstrate distinguishable steps, indicating that the accuracy of  moderate-coherence sensing surpasses 1 nm. Notably, at longer distances (Fig. 8c), the signal exhibits some noise.

To achieve an absolute accuracy of one nanometer, the cavity length under measurement should not vary by more than one nanometer during the 50 μs measurement time. This equates to a maximum speed of 20 μm/s. Increasing the piezo frequency may enhance measurable speed under specific conditions.

To evaluate reproducibility, the three distances were each measured 50 times in succession. Results in Fig. 8d–f show standard deviations: For 26.533 μm, *σ*_MCS_ < ± 0.3 nm; for 62.286 μm, *σ*_MCS_ < ± 0.3 nm; and for the largest distance, 102.782 μm, *σ*_MCS_ < ± 0.6 nm. The increased error for longer cavity lengths is likely due to reduced cavity visibility, leading to smaller mid-fringe increments (see Fig. 6). This reduction contributes to increased error in mid-fringe detection, making offset determination more challenging.

In summary, the investigated prototype of moderate-coherence sensing demonstrates accuracy and reproducibility better than 1 nm across a 120 μm measuring range. The corresponding relative length measurement error is thus less than 0.0000083.

## Discussion

In this study, we have presented the concept of moderate-coherence sensing and demonstrated its application in high-accuracy length measurements. The moderate-coherence sensing technique leverages the interference signal of a cavity along with the visibility parameter to achieve high accuracy within a wide measurement range and high measurement bandwidth >20 kHz. Our experimental implementation showcases the effectiveness of this approach in achieving sub-nanometer accuracy in length measurement over a range of 120 μm.

The experimental results confirmed the theoretical calculations, demonstrating excellent agreement between the measured visibility and the calculated visibility. This agreement validates the dominant effect of coherence modulation in the experiment, suggesting that other factors such as misalignment and laser divergence can be neglected.

Moreover, we explored the real-time measurement capabilities of moderate-coherence sensing. By scanning the cavity length over half a wavelength using a ring piezo, we were able to determine the actual cavity length through precise measurement of the mid-fringe position combined with visibility calculations. This real-time mode offers a fast and accurate measurement technique, enabling applications that require dynamic and rapid length monitoring.

The versatility of moderate-coherence sensing extends beyond length measurements. Any measurement that can be accessed using cavity length variations can be potentially measured using this technique. Temperature sensors, acceleration sensors, pressure sensors, and other sensors relying on the change in cavity length can benefit from the high accuracy and wide range offered by moderate-coherence sensing.

In conclusion, moderate-coherence sensing represents a powerful and promising approach for high-accuracy measurement applications. The combination of a sensitive interference signal and visibility analysis allows for precise measurements with a wide measurement range and high bandwidth. The experimental results demonstrate the practical feasibility and accuracy of this technique in length measurements and future sensors. The potential for further advancements and applications in various sensing fields makes moderate-coherence sensing a valuable tool for future research and technological developments.

### Supplementary information

Supplementary Information

## Data Availability

The authors declare that the data supporting the findings of this study are available within the paper.

## Code availability

Computer codes used in the current study are available from the corresponding author upon reasonable request.
