# Supplementary material for: Moderate-coherence sensing with optical cavities: ultra-high accuracy meets ultra-high measurement bandwidth and range
Source: Commun Eng. 2024 Jan 25;3:17. doi: 10.1038/s44172-024-00164-w (PMC10955870; doi:10.1038/s44172-024-00164-w)
Supplement: Supplementary file 1 — Supplementary Information [file 44172_2024_164_MOESM1_ESM.pdf]

SUPPLEMENTARY MATERIAL: Moderate-coherence sensing with optical cavities: ultra-high accuracy meets ultra-high measurement bandwidth and range

J. Dickmann*,[1,2,3] L. Shelling Neto,[1,2,3] S. Sauer,[1,2,3] and S. Kroker[2,3,4]

[1)] *CAVITY technologies UG (haftungsbeschränkt), Wilhelmsgarten 3, 38100 Braunschweig, Germany*

[2)] *Technical University of Braunschweig, Institute for Semiconductor Technology, Hans-Sommer-Str. 66, 38106 Braunschweig, Germany*

[3)] *Laboratory for Emerging Nanometrology, Langer Kamp 6a/b, 38106 Braunschweig, Germany*

[4)] *Physikalisch-Technische Bundesanstalt, Bundesallee 100, 38116 Braunschweig, Germany*

(*Electronic mail: j.dickmann@tu-braunschweig.de)

(Dated: 10 January 2024)

TABLE I: Mid-fringe lookup table: mid-fringe position's dependency on visibility for both negative and positive mid-fringe increments.

Visibility	Mid-fringe position, neg. in µm	Mid-fringe position, pos. in µm
0.999896	0.654005	0.896007
0.999583	1.429012	1.671014
0.999062	2.204018	2.446020
0.998335	2.979025	3.221027
0.997404	3.753031	3.997033
0.996271	4.528038	4.772040
0.994939	5.303044	5.547046
0.993412	6.078051	6.322053
0.991692	6.853057	7.097059
0.989786	7.627064	7.873066
0.987696	8.402070	8.648072
0.985429	9.177076	9.423079
0.982988	9.951083	10.199085
0.980381	10.726089	10.974091
0.977611	11.500096	11.749098
0.974686	12.275102	12.525104
0.971611	13.050109	13.300111
0.968391	13.824115	14.076117
0.965034	14.599122	14.851124
0.961544	15.373128	15.627130
0.957929	16.148135	16.402137
0.954194	16.922141	17.178143

Continued on next page

TABLE I: Mid-fringe lookup table: mid-fringe position's dependency on visibility for both negative and positive mid-fringe increments. (Continued)

Visibility	Mid-fringe position, neg. in µm	Mid-fringe position, pos. in µm
0.950345	17.696147	17.953150
0.946387	18.471154	18.729156
0.942327	19.245160	19.504163
0.938171	20.020167	20.280169
0.933923	20.794173	21.056175
0.929589	21.568180	21.831182
0.925175	22.343186	22.607188
0.920685	23.117193	23.383195
0.916123	23.891199	24.158201
0.911496	24.665206	24.934208
0.906807	25.440212	25.710214
0.902061	26.214218	26.486221
0.897261	26.988225	27.261227
0.892412	27.762231	28.037234
0.887517	28.537238	28.813240
0.882580	29.311244	29.589247
0.877604	30.085251	30.364253
0.872593	30.859257	31.140260
0.867550	31.633264	31.916266
0.862477	32.408270	32.692272
0.857377	33.182277	33.468279
0.852253	33.956283	34.243285

Continued on next page

TABLE I: Mid-fringe lookup table: mid-fringe position's dependency on visibility for both negative and positive mid-fringe increments. (Continued)

Visibility	Mid-fringe position, neg. in μm	Mid-fringe position, pos. in μm
0.847108	34.730289	35.019292
0.841942	35.504296	35.795298
0.836759	36.279302	36.571305
0.831561	37.053309	37.347311
0.826349	37.827315	38.122318
0.821125	38.601322	38.898324
0.815890	39.375328	39.674331
0.810647	40.149335	40.450337
0.805395	40.923341	41.226344
0.800138	41.698347	42.002350
0.794875	42.472354	42.777356
0.789607	43.246360	43.553363
0.784336	44.020367	44.329369
0.779063	44.794373	45.105376
0.773787	45.568380	45.881382
0.768511	46.343386	46.657389
0.763233	47.117393	47.432395
0.757956	47.891399	48.208402
0.752678	48.665406	48.984408
0.747401	49.439412	49.760415
0.742124	50.214418	50.536421
0.736848	50.988425	51.311428

Continued on next page

TABLE I: Mid-fringe lookup table: mid-fringe position's dependency on visibility for both negative and positive mid-fringe increments. (Continued)

Visibility	Mid-fringe position, neg. in µm	Mid-fringe position, pos. in µm
0.731573	51.762431	52.087434
0.726299	52.536438	52.863441
0.721026	53.310444	53.639447
0.715753	54.085451	54.415453
0.710481	54.859457	55.190460
0.705210	55.633464	55.966466
0.699939	56.407470	56.742473
0.694669	57.182477	57.518479
0.689398	57.956483	58.293486
0.684126	58.730489	59.069492
0.678854	59.504496	59.845499
0.673580	60.279502	60.620505
0.668305	61.053509	61.396512
0.663028	61.827515	62.172518
0.657749	62.602522	62.947525
0.652467	63.376528	63.723531
0.647181	64.150535	64.499537
0.641892	64.925541	65.274544
0.636599	65.699547	66.050550
0.631302	66.473554	66.826557
0.626000	67.248560	67.601563
0.620693	68.022567	68.377570

Continued on next page

TABLE I: Mid-fringe lookup table: mid-fringe position's dependency on visibility for both negative and positive mid-fringe increments. (Continued)

Visibility	Mid-fringe position, neg. in µm	Mid-fringe position, pos. in µm
0.615380	68.797573	69.152576
0.610062	69.571580	69.928583
0.604737	70.346586	70.703589
0.599407	71.120593	71.479596
0.594070	71.894599	72.254602
0.588727	72.669606	73.030609
0.583377	73.443612	73.805615
0.578021	74.218618	74.581622
0.572658	74.993625	75.356628
0.567289	75.767631	76.132634
0.561914	76.542638	76.907641
0.556532	77.316644	77.683647
0.551145	78.091651	78.458654
0.545752	78.865657	79.233660
0.540353	79.640664	80.009667
0.534950	80.415670	80.784673
0.529542	81.189677	81.560680
0.524131	81.964683	82.335686
0.518715	82.739689	83.110693
0.513297	83.513696	83.885699
0.507876	84.288702	84.661706
0.502454	85.063709	85.436712

Continued on next page

TABLE I: Mid-fringe lookup table: mid-fringe position's dependency on visibility for both negative and positive mid-fringe increments. (Continued)

Visibility	Mid-fringe position, neg. in µm	Mid-fringe position, pos. in µm
0.497030	85.838715	86.211718
0.491605	86.612722	86.987725
0.486181	87.387728	87.762731
0.480757	88.162735	88.537738
0.475335	88.937741	89.312744
0.469915	89.711748	90.088751
0.464498	90.486754	90.863757
0.459084	91.261761	91.638764
0.453675	92.036767	92.413770
0.448271	92.811773	93.188777
0.442873	93.585780	93.964783
0.437481	94.360786	94.739789
0.432097	95.135793	95.514796
0.426721	95.910799	96.289802
0.421354	96.685806	97.064809
0.415997	97.460812	97.839815
0.410650	98.234819	98.615822
0.405315	99.009825	99.390828
0.399993	99.784832	100.165835
0.394683	100.559838	100.940841
0.389387	101.334844	101.715848
0.384106	102.109851	102.490854

Continued on next page

TABLE I: Mid-fringe lookup table: mid-fringe position's dependency on visibility for both negative and positive mid-fringe increments. (Continued)

Visibility	Mid-fringe position, neg. in µm	Mid-fringe position, pos. in µm
0.378840	102.884857	103.265861
0.373591	103.659864	104.040867
0.368359	104.434870	104.816873
0.363146	105.208877	105.591880
0.357951	105.983883	106.366886
0.352776	106.758890	107.141893
0.347622	107.533896	107.916899
0.342489	108.308903	108.691906
0.337379	109.083909	109.466912
0.332293	109.858915	110.241919
0.327230	110.633922	111.016925
0.322193	111.408928	111.792932
0.317181	112.183935	112.567938
0.312197	112.958941	113.342945
0.307240	113.733948	114.117951
0.302312	114.507954	114.892957
0.297414	115.282961	115.667964
0.292545	116.057967	116.442970
0.287709	116.832974	117.217977
0.282904	117.607980	117.993983
0.278133	118.382987	118.768990
0.273396	119.157993	119.543996
